# Supplementary material for: Oncogenic KRAS is dependent upon an EFR3A-PI4KA signaling axis for potent tumorigenic activity
Source: Nat Commun. 2021 Sep 9;12:5248. doi: 10.1038/s41467-021-25523-5 (PMC8429657; doi:10.1038/s41467-021-25523-5)
Supplement: Supplementary file 1 — Supplementary Information [file 41467_2021_25523_MOESM1_ESM.pdf]

SUPPLEMENTARY INFORMATION

Oncogenic KRAS is dependent upon an EFR3A-PI4KA signaling axis for potent tumorigenic activity

Hema Adhikari[1], Walaa E. Kattan[2,3], Shivesh Kumar[4], Pei Zhou[4], John F. Hancock[2,3,*] and Christopher M. Counter[1,*]

[1] Department of Pharmacology & Cancer Biology, Duke University, Durham, North Carolina, USA
[2] Integrative Biology & Pharmacology, McGovern Medical School, University of Texas Health Science Center, Houston, Texas, USA
[3] The University of Texas MD Anderson Cancer Center UTHealth Graduate School of Biomedical Sciences at Houston, Houston, Texas, USA
[4] Department of Biochemistry, Duke University, Durham, North Carolina, USA
[*] corresponding authors

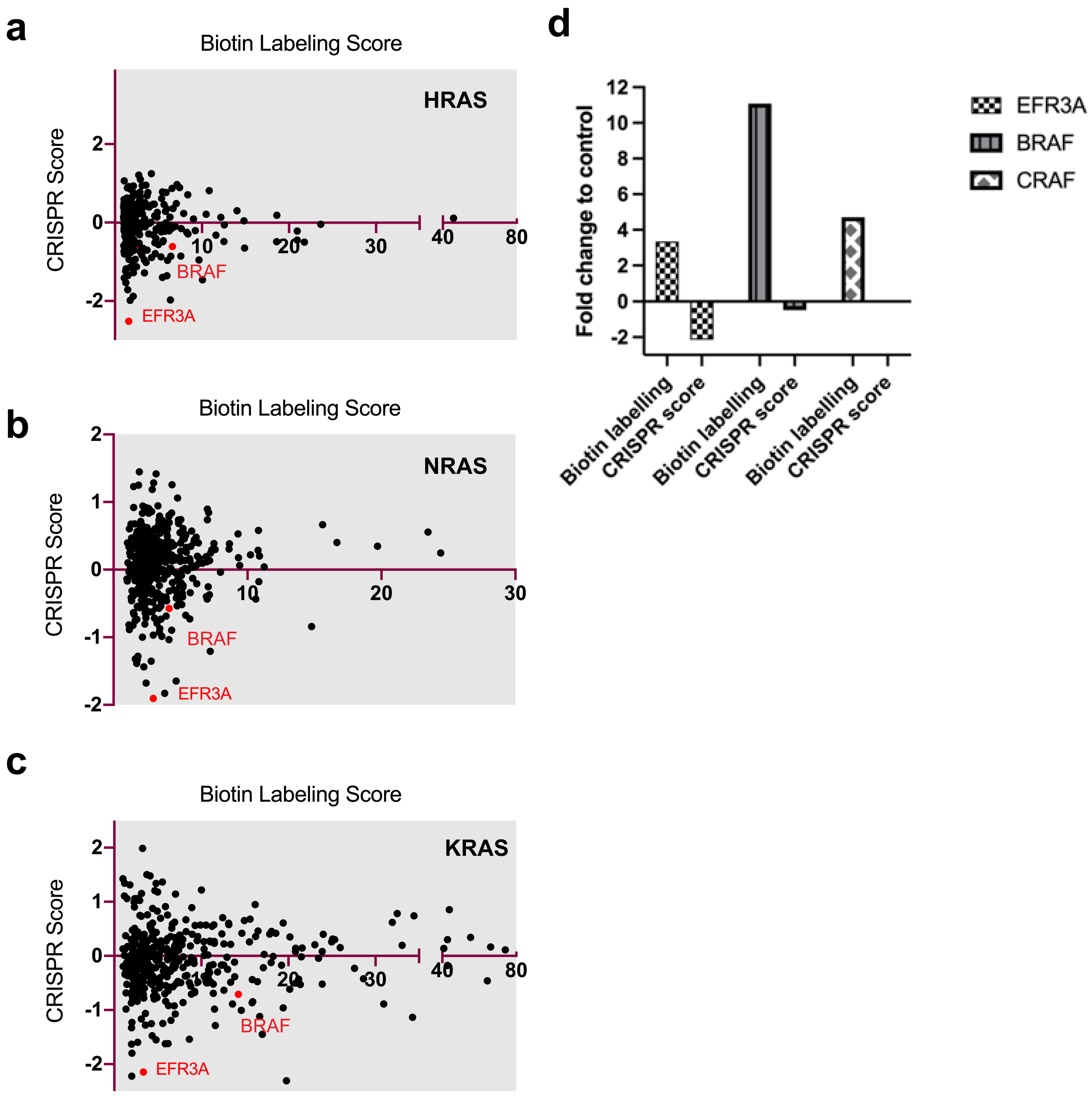

Supplementary Fig. 1. Identification of EFR3A from a RAS BioID/CRISPR Cas9 screen. a-c) XY scatter plot of the interactomes **a)** HRAS, **b)** NRAS and **c)** KRAS comparing fold change scores of biotin labeling with CRISPR gene enrichment, *p*- value ≤0.05, one-sided student *t*-test. **d)** Comparison of RAF (CRAF and BRAF) and EFR3A biotin labeling with oncogenic KRAS (BirA*-KRAS^{G12V}) and comparison with CRISPR depletion score of *CRAF, BRAF* and *EFR3A* in oncogenic KRAS (KRAS^{G12V}) HEK-HT transformed cells.

Supplementary_Fig.2

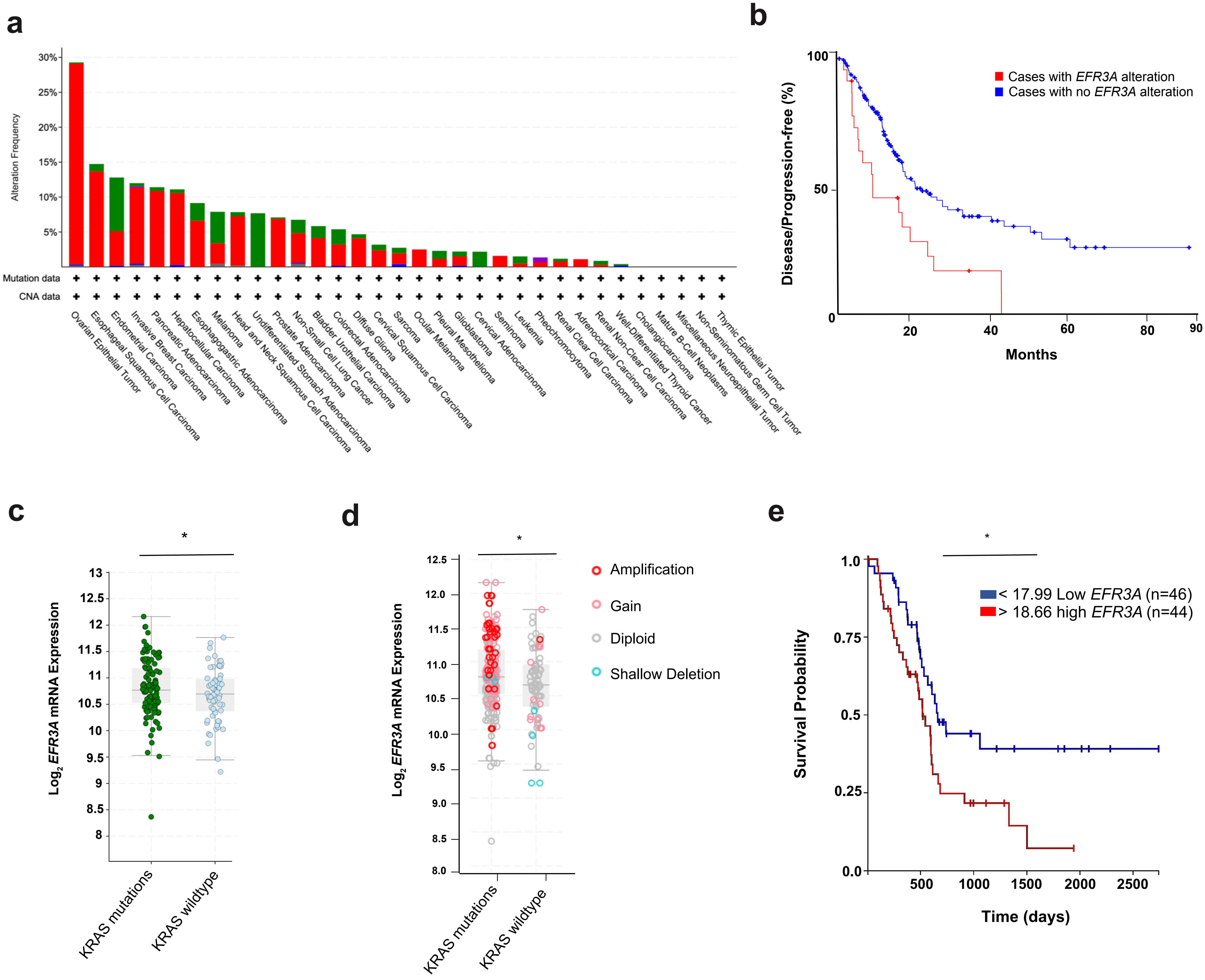

Supplementary Fig. 2. Genomic alterations of *EFR3A* in different cancers. a) The frequency that the *EFR3A* gene exhibits amplification and copy number variation (red bar), a point mutation (green bar), deletion (blue bar), or multiple alterations (grey bar) in different cancers from the cBiportal dataset. **b)** Kaplan-Meier estimate of disease-free survival of pancreatic adenocarcinoma cases with either *EFR3A* alteration or no alteration. Altered cases: 19/25 recurred progressed with a total median months disease free (9.57 months). Unaltered cases: 88/185 recurred progressed with a total median months disease free (20.38 months), p=1.088e-4. **c)** A boxplot of the $\log_2$ mRNA expression of *EFR3A* mRNA in 184 pancreatic tumors with wild-type *KRAS* versus with a missense mutation in *KRAS* by gene expression analysis of the TCGA pancreatic adenocarcinoma 2018. *KRAS* mutant sample, max (12.2), min (9.6), center (10.7); wildtype sample, max (11.7), min (9.4), center (10.6). **d)** Dataset from c) overlaid with the status of the *EFR3A* gene (diploid: both alleles are wild-type, shallow deletion: a heterozygous deletion, gain: a low level gain of a few additional copies that is broad, amplification: a high level amplification of additional copies that is focal). *KRAS* mutant sample max (12.2), min (9.6), center (10.7); wildtype sample max (11.7), min (9.4), center (10.6). **e)** A Kaplan-Meier plot of overall survival of pancreatic adenocarcinoma cases with high (define, n=44) versus low (define, n=46) *EFR3A* mRNA expression as determined from 5 pancreatic cancer datasets (ICGC, Nature 2012; TCGA PanCancer Atlas; UTSW Pancreatic Cancer; TCGA Firehose Legacy; QCMG, Nature 2016), p= 0.01504. Significance values calculated by one-way ANOVA analysis; *$p < 0.05$.

Supplementary_Fig.3

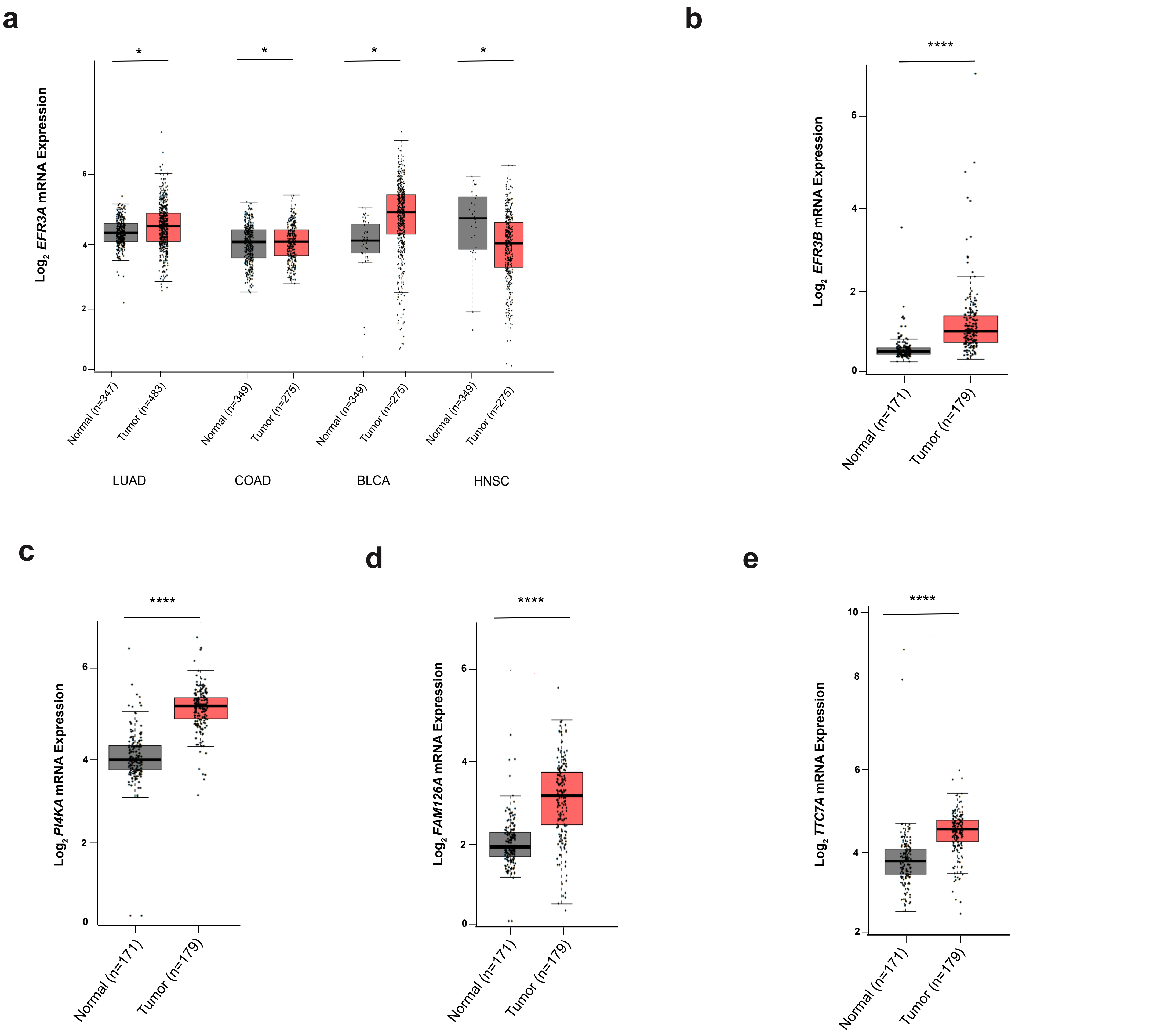

Supplementary Fig. 3. *EFR3A, EFR3B, PI4KA, FAM126A and TTC7A* mRNA levels in cancer. a) GEPIA analysis of *EFR3A* mRNA levels in lung cancer (LUAD), normal sample max (5.1), min (3.5), center (4.3); tumor sample max (6.0), min (2.9), center (4.3), colorectal cancer (COAD), normal sample max (5.2), min (2.7), center (4.1); tumor sample max (5.3), min (2.8), center (4.1), bladder cancer (BLCA), normal sample max (5.0), min (3.6), center (4.2); tumor sample max (6.5), min (2.3), center (4.9), head and neck cancer (HNSC) normal sample max (5.8), min (2.1), center (4.7); tumor sample max (6.2), min (1.6), center (4.1), dataset in tumor and normal tissue. **b-e)** A boxplot of the $\log_2$ mRNA levels of, **b)** *EFR3B*, normal sample max (0.8), min (0.3), center (0.5); tumor sample max (2.4), min (0.4), center (1.1), **c)** *PI4KA*, normal sample max (4.8), min (3.1), center (4.0); tumor sample max (5.9), min (4.2), center (5.1), **d)** *FAM126A*, normal sample max (3.2), min (1.1), center (1.9); tumor sample max (4.8), min (0.3), center (3.2), and **e)** *TTC7A* normal sample max (4.7), min (2.6), center (3.8); tumor sample max (5.3), min (3.4), center (4.5), in 179 pancreatic adenocarcinoma versus 171 normal tissues based on GEPIA analysis of the PAAD dataset. n=number of samples analyzed. Significance values calculated by one-way ANOVA analysis; *p < 0.05, and ****p < 0.0001.Percentiles in the box plots pre-set at 50% (higher) and 50% (lower).

Supplementary_Fig.4

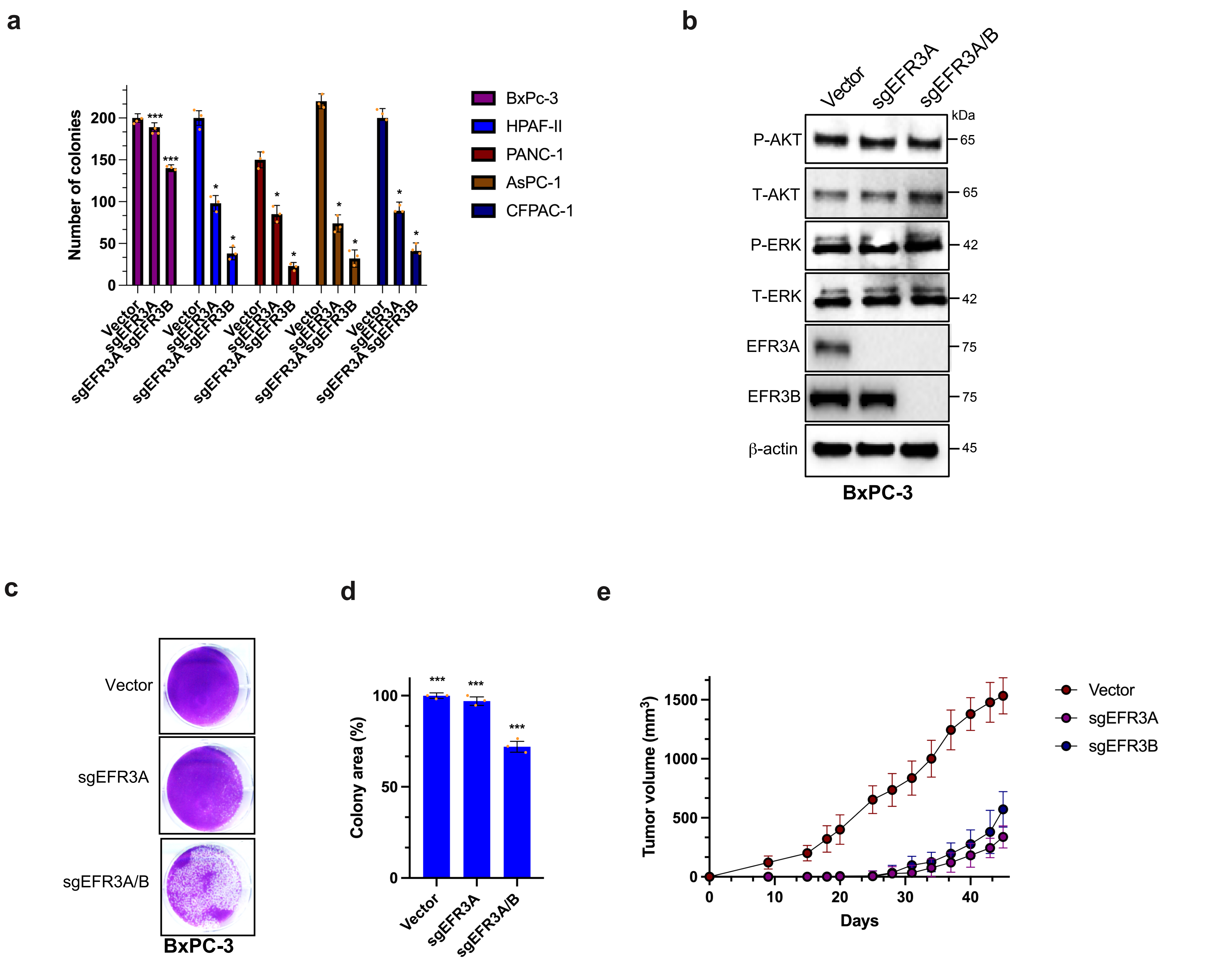

Supplementary Fig. 4. Inactivation of EFR3A inhibits KRAS signaling in transformed and tumorigenic growth. a) Mean ± SD colony formation as assessed by soft agar growth assay in the indicated cells. Calculated *p* values of BxPc3; 0.00047 (*sgEFR3A)*, 0.00051 (*sgEFR3A/B)*, HPAF-II; 0.048(*sgEFR3A)*, 0.035 (*sgEFR3A/B)*, PANC-1; 0.047 (*sgEFR3A)*, 0.023 (*sgEFR3A/B)*, AsPc-1; 0.045 (*sgEFR3A)*, 0.048 (*sgEFR3A/B)*, CFPAC-1; 0.047(*sgEFR3A)*, 0.05 (s*gEFR3A/B)*. **b)** Levels of phosphorylated (P) and/or total (T) ERK, AKT, EFR3A, EFR3B, and actin (as a loading control), as assessed by immunoblot analysis. **c)** Transformed growth as assessed by crystal violet staining in BxPC-3 human pancreatic adenocarcinoma (*KRASWT*) cell line transduced with indicated sgRNAs. **d)** Mean ± SD of colony area of cells from c. Calculated p values of 0.00047 (*sgEFR3A)*, 0.00051 (*sgEFR3A/B*). **e)** Mean ± SD tumor volume (mm^3) versus time of HPAF-II cells stably infected with a lentivirus(es) encoding Cas9 alone (vector) or *EFR3A sgRNA* (*sgEFR3A-2*) and *EFR3B sgRNA (sgEFR3B)* injected into five mice each. Immunoblots are representative of three biological replicates. Cell viability assays are representative of three biological replicates assayed in triplicate. Significance values for xenograft assay was calculated by one-way ANOVA test and for xenograft (**$p < 0.01$), for cell viability and for crystal violet assay quantitation one-sided student t test was used; *$p < 0.05$ and ***$p < 0.001$. Full-length gel image provided in Supplementary Fig.17.

Supplementary_Fig.5

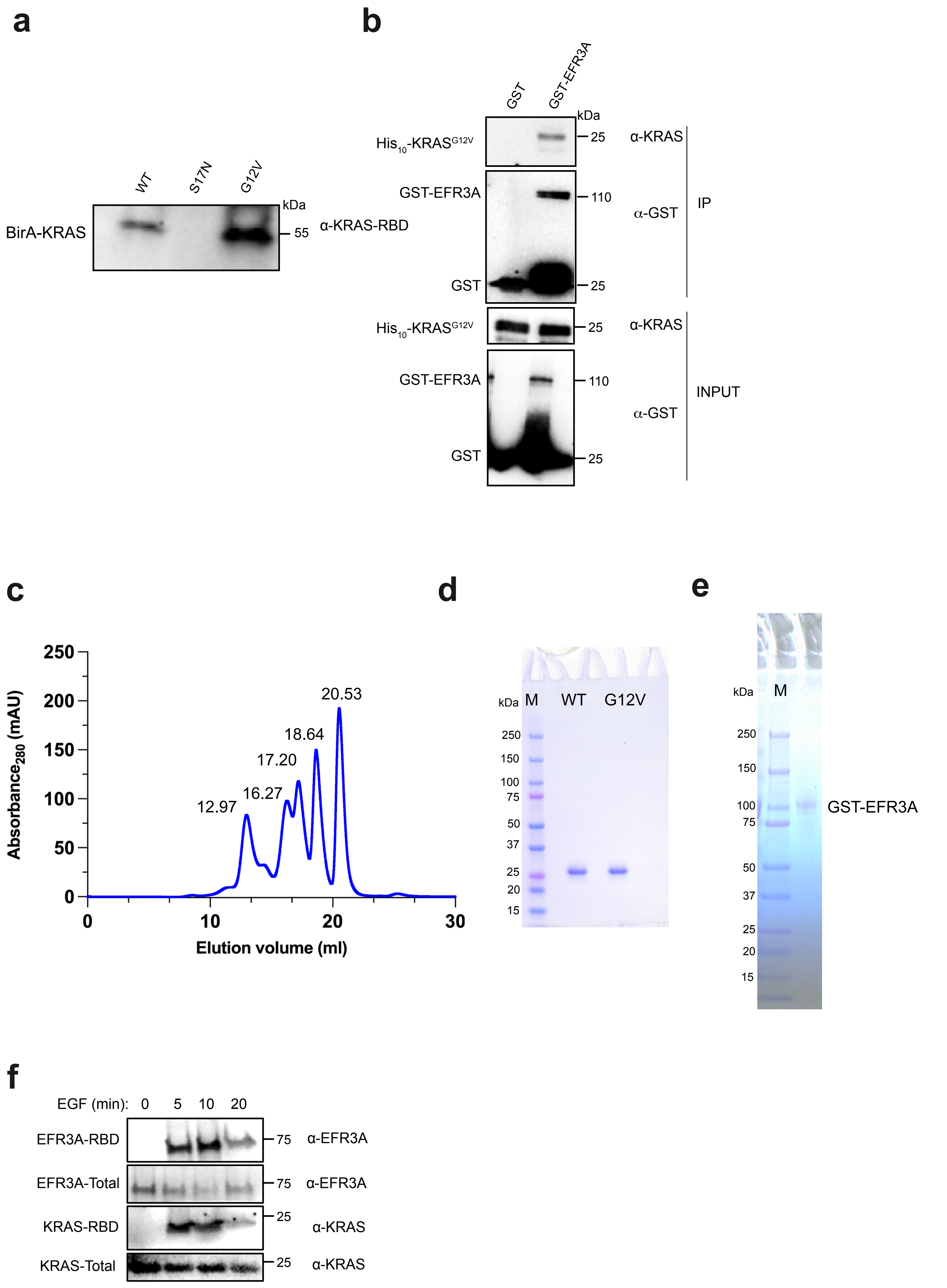

Supplementary Fig. 5. Validation of EFR3A-KRAS interaction. a) GTP loading of the respective recombinant KRAS proteins as assessed by RBD pull-down assay using 293T cells stably expressing myc epitope tagged-BirA-KRAS^{WT}, BirA-KRAS^{G12V} and BirA-KRAS^{S17N}. **b)** Levels of oncogenic KRAS associating with EFR3A in vitro, as assessed by immunoprecipitating (IP) recombinant GST or GST-EFR3A using GST antibody in the presence of recombinant His$_{10}$-KRAS^{G12V}, followed by immunoblot with an anti-KRAS antibody to detect co-immunoprecipitated (CO-IP) KRAS. INPUT and IP levels of the indicated proteins serve as controls. **c)** Size-exclusion chromatography elution profile of Biorad protein standard depicting the elution volume (EV) in ml corresponding to the molecular weight. The standard components include thyroglobulin; MW,670 kDa, EV12.97, globulin; MW,158 kDa, EV16.27, ovalbumin; MW,44 kDa EV 17.20, myoglobin; MW,17 kDa EV18.64; vitamin B12; MW,1.35 kDa EV 20.53. **d,e)** First peak fractions of **d)** recombinant His$_{10}$-KRAS^{WT} and His$_{10}$-KRAS^{G12V} or **e)** recombinant GST-EFR3A after size exclusion chromatography were concentrated and resolved on SDS-PAGE gel. **f)** Levels of endogenous EFR3A associating with endogenous KRAS upon EGF stimulation over time as assessed by RBD pull-down assay in BxPC-3 cells. Immunoblots in Fig. 5a,b and SDS PAGE gels in Fig. 5d,e are representative of three biological replicates and immunoblots in Fig. 5f is representative of two biological resplicates. Full-length gel image provided in Supplementary Fig.17.

Supplementary_Fig.6

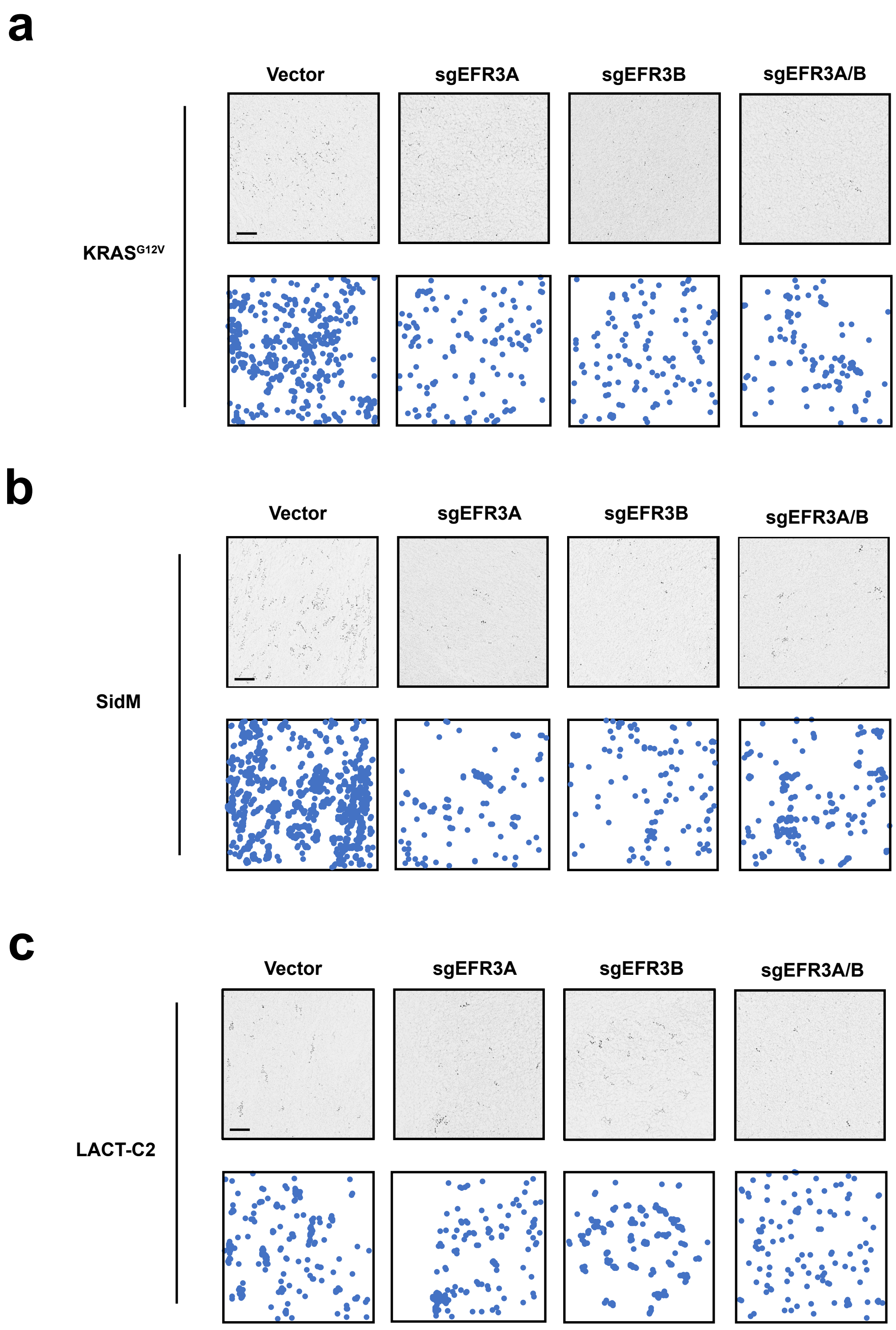

Supplementary Fig. 6. Representative EM images. **a-c)** Immunogold-labeled plasma membrane sheets of the data quantified in Fig. 6f,g and Fig. 8b,d. Plasma membrane sheets were labeled with anti-GFP antibodies coupled directly to 4.5-nm gold particles and visualized by EM, Scale bar: 100nM. The (x,y) coordinates of each identified gold particle on the plasma membrane sheet are plotted to scale for visualization of the gold pattern. P4M-SidM (PI(4)P probe), and LACT-C2 (PS probe). TEM analysis is based on 30 images. Bootstrap tests were used to assess significance of nanoclustering. Bootstrap tests were used to examine for statistical differences between replicated point patterns and statistical significance was evaluated against 1000 bootstrap samples.

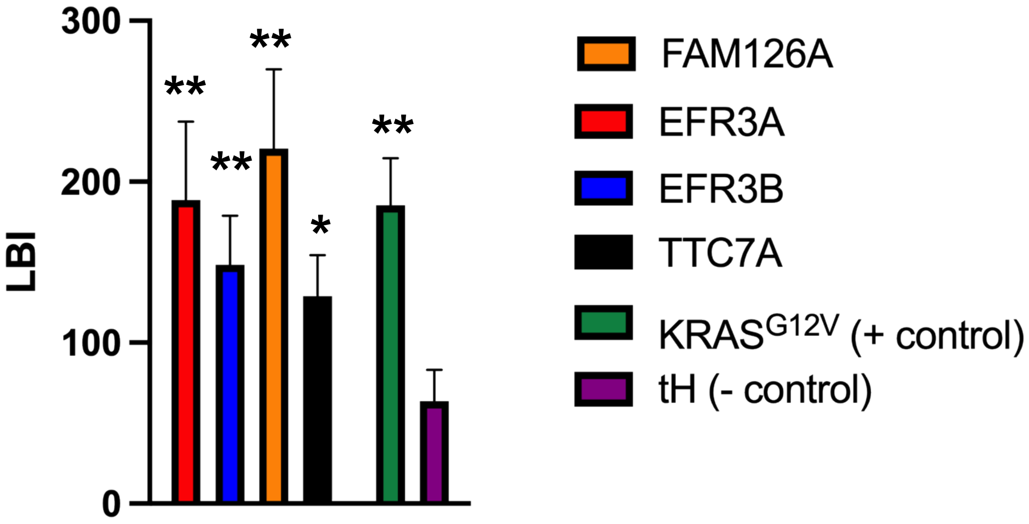

Supplementary Fig. 7. Co-clustering index (LBI). Mean ± SEM of the gold particles per 1µm^2 of plasma membrane sheets calculated from gold-conjugated, anti-GFP antibody and anti-RFP antibody in EM micrographs of plasma membrane sheets from human 293T cells co-expressing GFP-KRAS^{G12V} and TTC7A-mCherry, FAM126A-mCherry, EFR3A-mCherry, or EFR3B-mCherry. TEM analysis was based on 30 images and bootstrap tests were used to examine for statistical differences between replicated point patterns and statistical significance was evaluated against 1,000 bootstrap samples. Statistics for immunogold particles (representative of nanoclustering) were calculated using non-parametric 2-tailed student's *t*-test. Significance values were calculated by the bootstrap tests; $*p < 0.05$, and $**p < 0.01$. Significance values were calculated by the two-sided bootstrap tests $*p< 0.02$ and $**p< 0.001$ compared to the tH negative control where it is known that tH does not co-cluster with KRAS. Specific significance values compared to tH negative control and KRAS^{G12V} positive control respectively of 0.001,1 (EFR3A), 0.001,1 (EFR3B), 0.001, 1 (FAM126A),0.02, 0.925 (TTC7A) and 0.001, 1 (KRAS^{G12V}).

Supplementary Fig._8

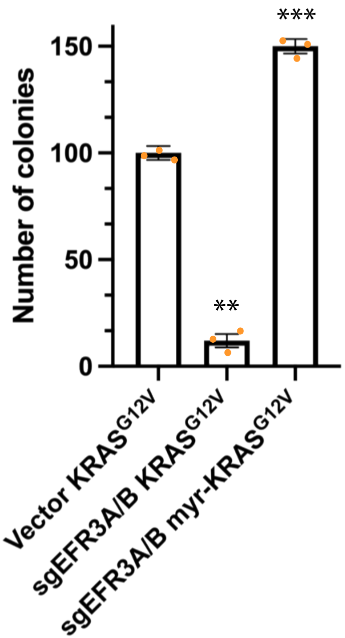

Supplementary Fig. 8. Myristoylated KRASG12V rescues 3D-transformed growth upon inactivation of EFR3A. Mean ± SD 3D-transformed growth as assessed by growth in soft agar and plotted as the number of colonies of human HEK-HT cells transformed by oncogenic $KRAS^{G12V}$ or myr-$KRAS^{G12V}$ (myristoylated) engineered to stably express Cas9 and a vector encoding no sgRNA (vector) or the indicated sgRNAs. Mean ± SD quantification of same cells as fig 7c. Soft agar assay is representative of three biological replicates assayed in triplicate. Significance values were calculated by one-way ANOVA test; $^{**}p < 0.01$ and $^{***}p < 0.001$. Specific significance values of 0.0035 (*sgEFR3A* $KRAS^{G12V}$), 0.00027 (*sgEFR3A/B myr-*$KRAS^{G12V}$).

Supplementary_Fig.9

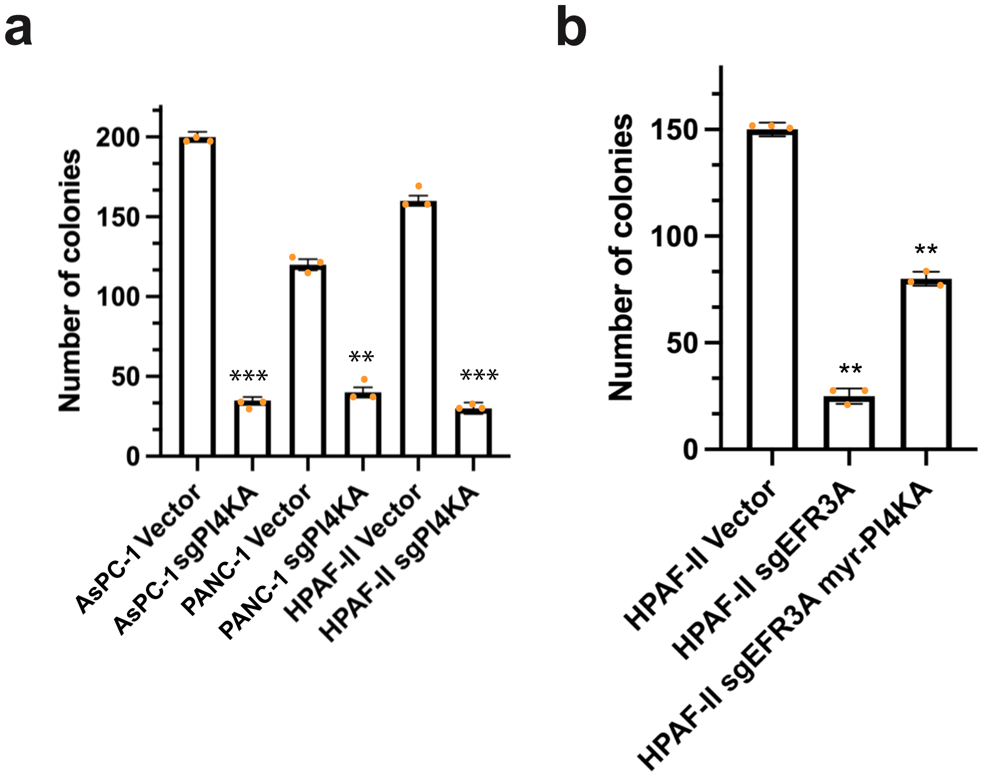

Supplementary Fig. 9. 3D transformed colony growth of the indicated cells. a,b) Mean ± SD colonies grown in soft agar of the indicated pancreatic adenocarcinoma cells stably transduced with a vector encoding Cas9 and no sgRNA (vector), **a)** *sgPI4KA*, **b)** or *sgEFR3A* alone or in conjunction with a vector encoding ectopic myr-PI4KA. Three biological replicates assayed in triplicate. Significance values were calculated by one-way ANOVA test; $^{**}p < 0.01$ and $^{***}p < 0.001$. Specific significance values of **a)** are 0.00016 (AsPc-1*sgPI4KA*), 0.0024 (PANC1*sgPI4KA)* and 0.00063 (HPAF-II*sgPI4KA*) and **b)** are 0.0035 (HPAF-II*sgEFR3A*) and 0.0047 (HPAF-II*sgEFR3A* myr-PI4KA).

Supplementary_Fig.10

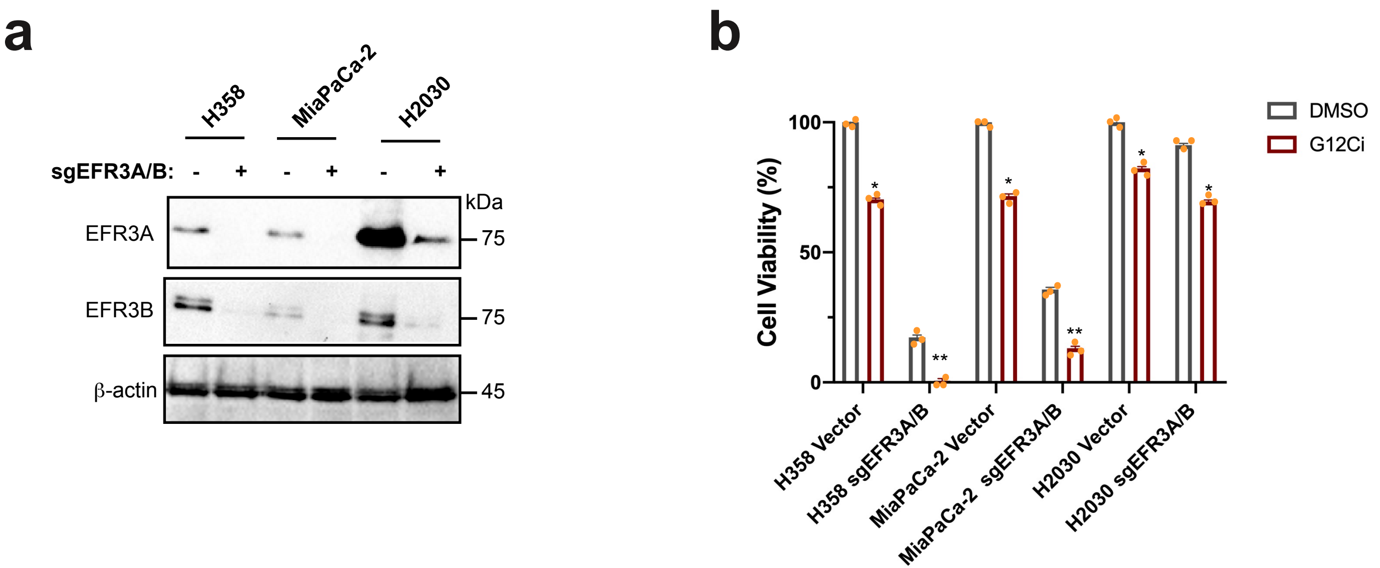

Supplementary Fig. 10. Dual inhibition of oncogenic RAS and EFR3A/B in *RAS*-mutant pancreatic cancer cells. a) Levels of EFR3A and EFR3B, as assessed by immunoblot analysis, and **b)** Mean ± SD cell viability, as assessed by CellTiter Glo, of human pancreatic cancer cells harboring oncogenic $KRAS^{G12C}$ and stably infected with a lentivirus(es) encoding Cas9 alone (vector) or independent sgRNAs targeting *EFR3A (sgEFR3A-2)* and *EFR3B (sgEFR3B-1)*. Immunoblot is representative of two biological replicates. Cell viability analysis is representative of 3 biological replicates assayed in triplicate. Significance values calculated by one-way ANOVA test; $*p < 0.05$ and $**p < 0.01$. Specific significance values are H358; 0.017 (Vector G12Ci), p=0.0026 (*sgEFR3A/B* G12Ci), MiaPaCa2; 0.021(Vector G12Ci), 0.0013(*sgEFR3A/B* G12Ci), H2030; 0.029 (Vector G12Ci), and 0.025 (*sgEFR3A/B* G12Ci).

Supplementary_Fig.11

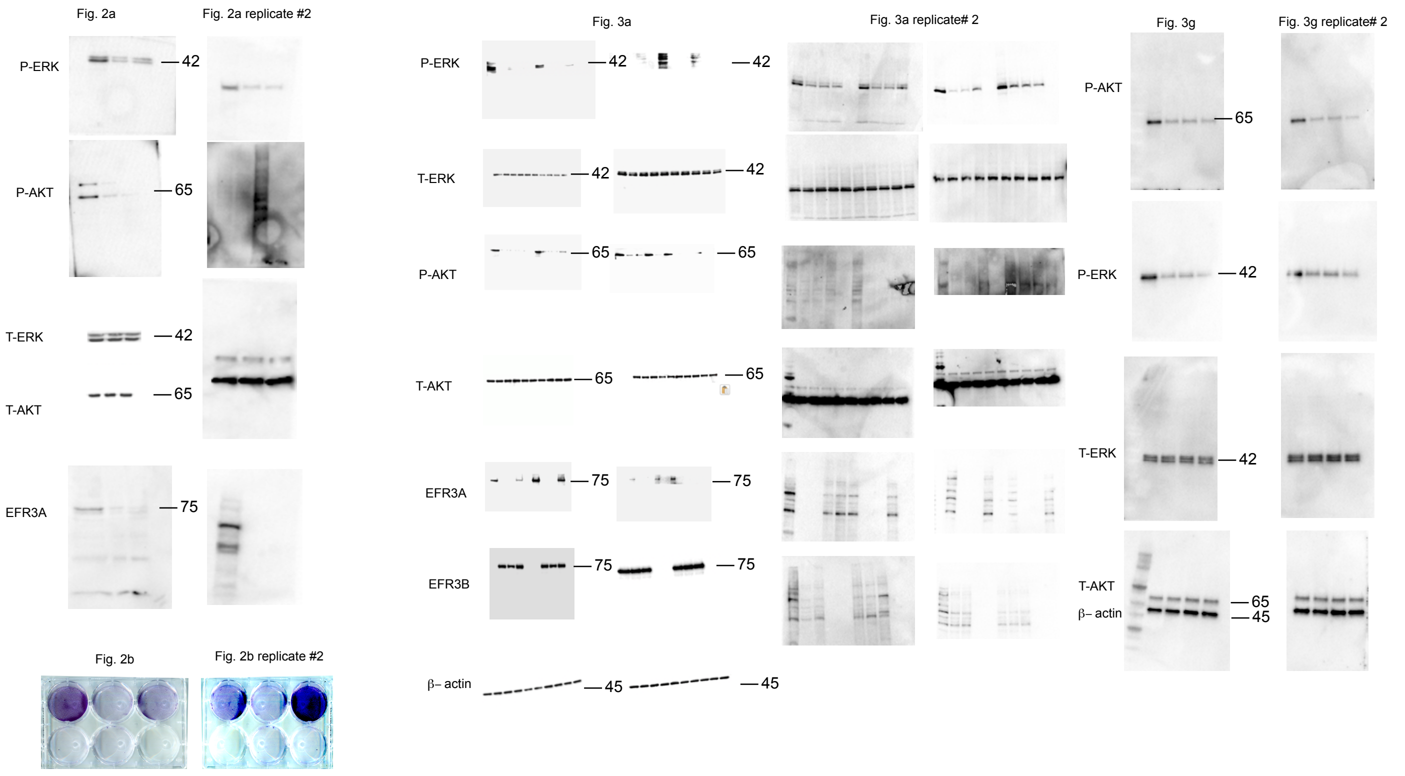

Supplementary Fig. 11. Uncropped gel images depicted in Figs. 2,3. and replicates

Supplementary_Fig.12

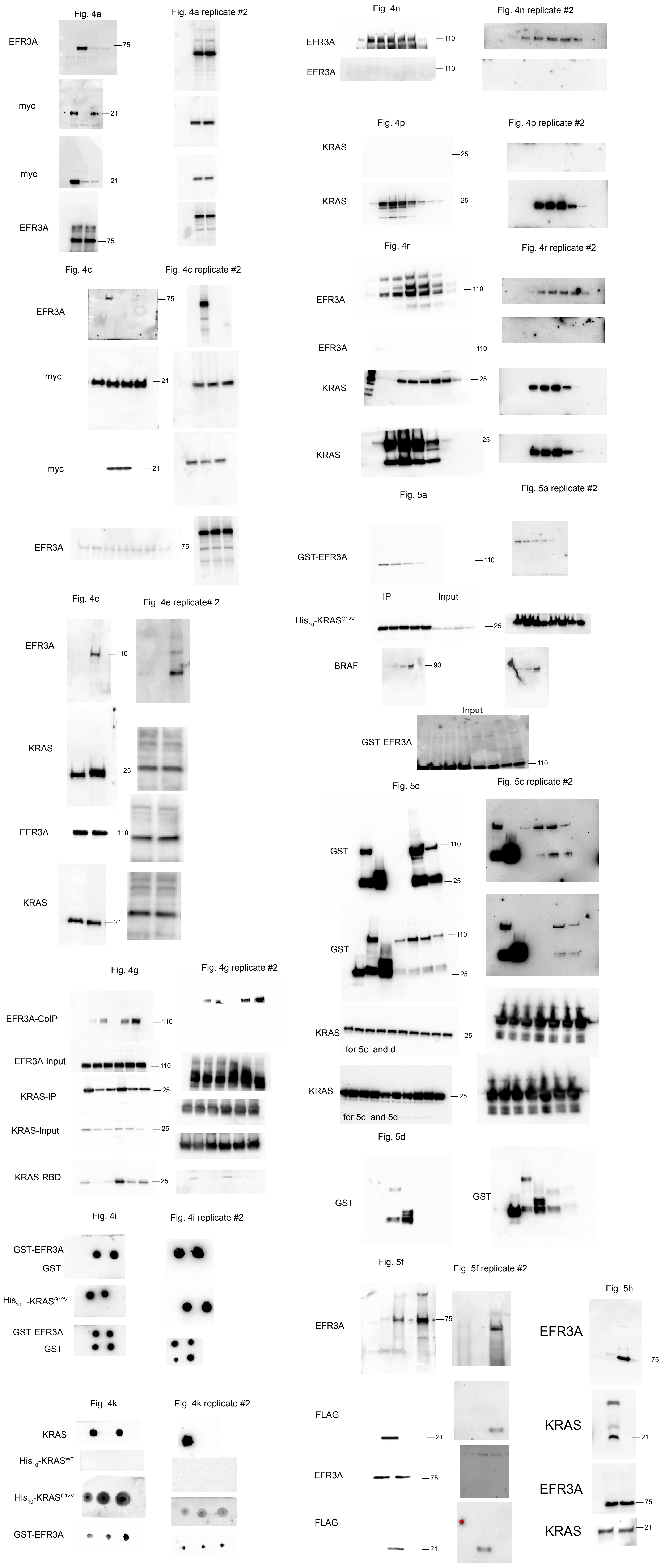

Supplementary Fig. 12. Uncropped gel images depicted in Figs. 4, 5 and replicates

Supplementary_Fig.13

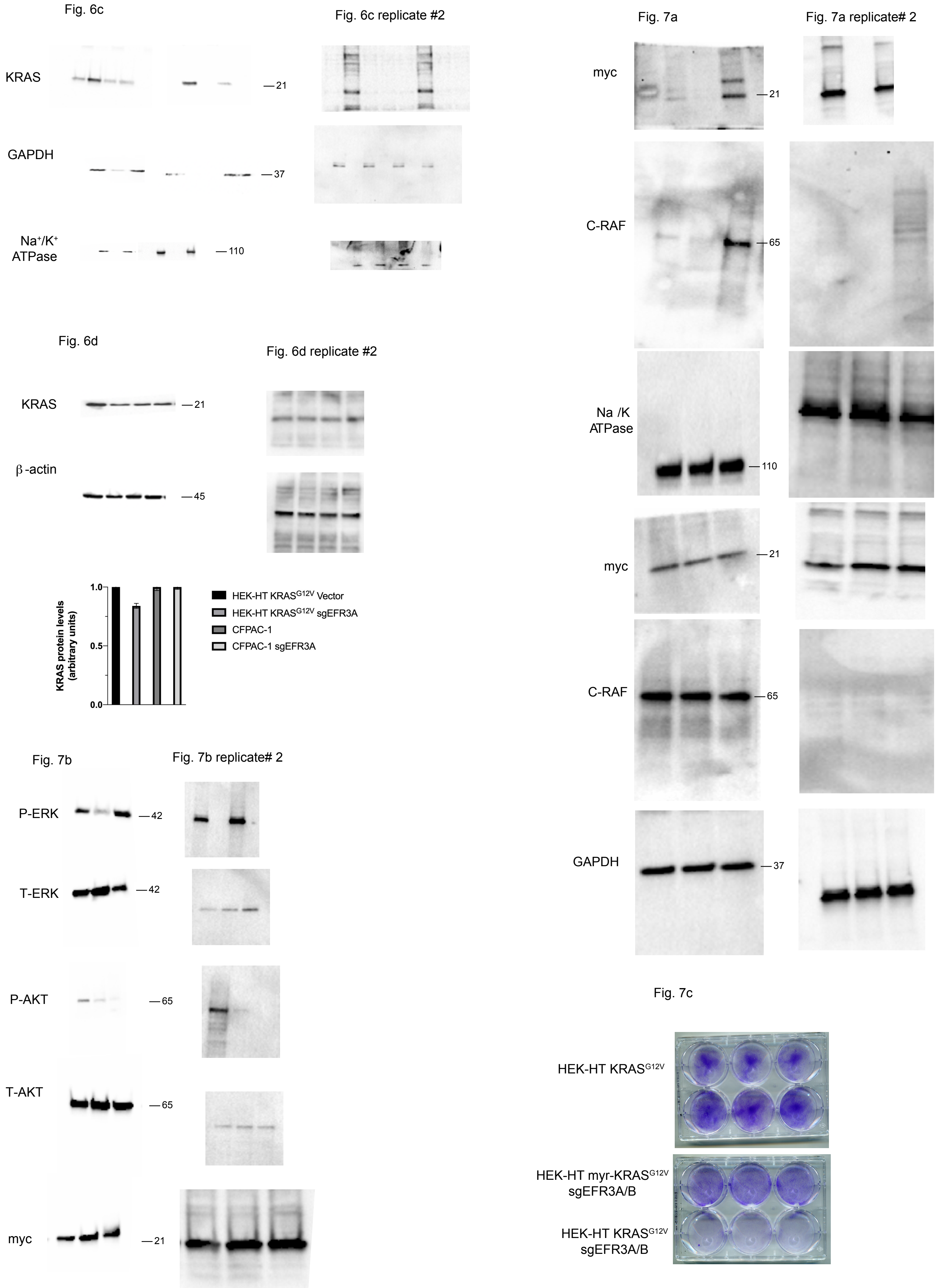

Supplementary Fig. 13. Uncropped gel images depicted in Figs.6,7 and replicates

Supplementary_Fig.14

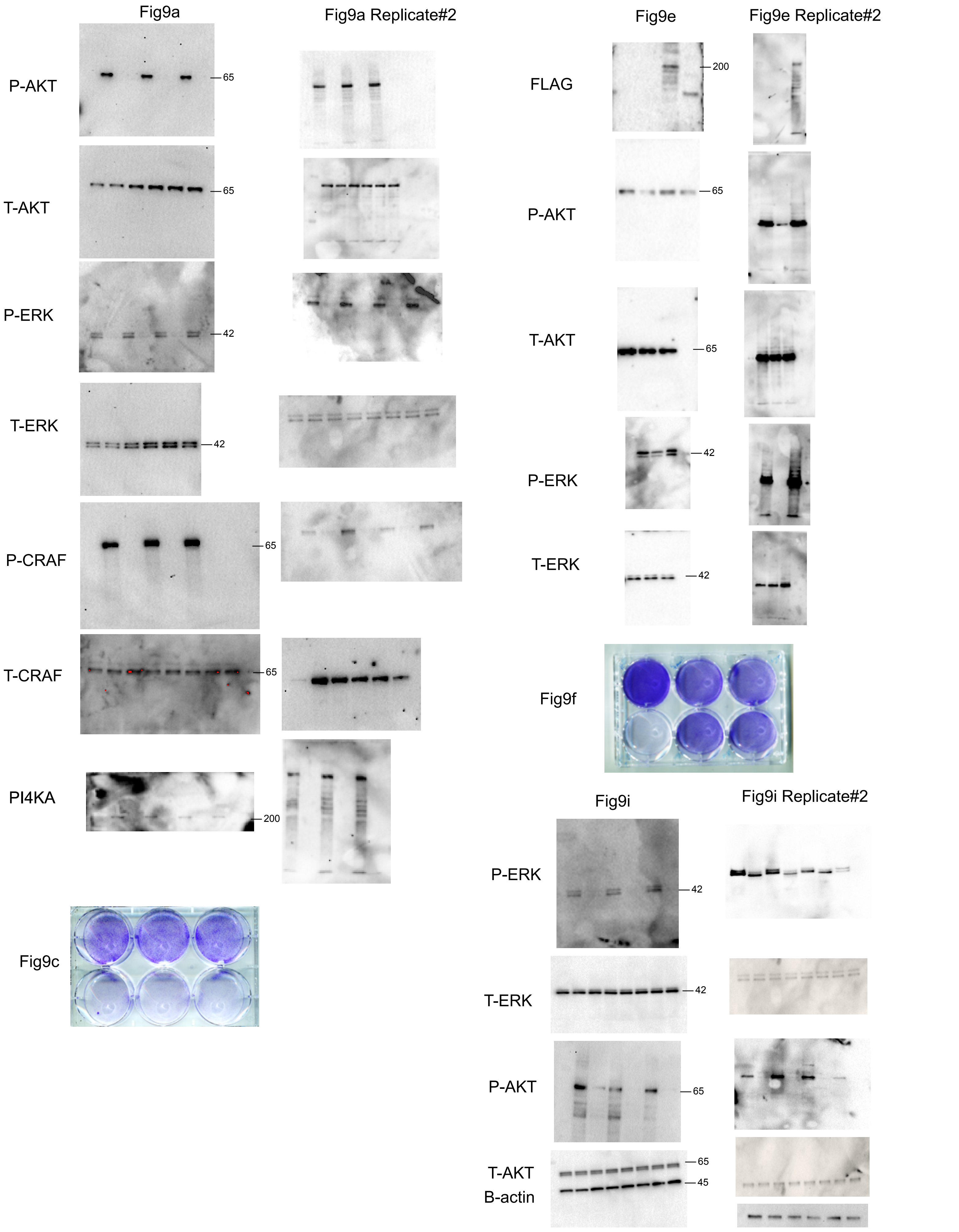

Supplementary Fig. 14. Uncropped gel images depicted in Fig. 9 and replicates

Supplementary_Fig.15

Fig. 10b

Fig. 10b replicate #2

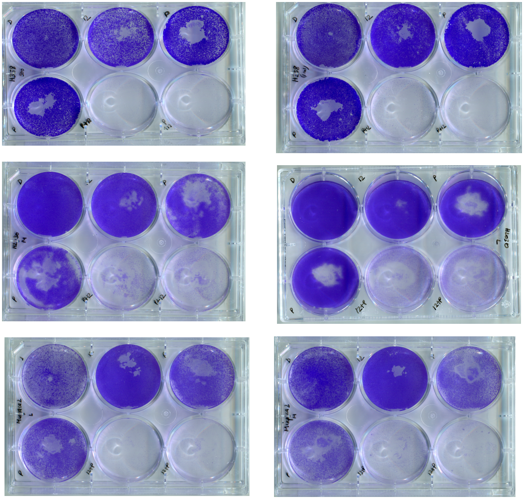

Supplementary Fig. 15. Uncropped gel images depicted in Fig. 10. and replicates

Supplementary_Fig.16

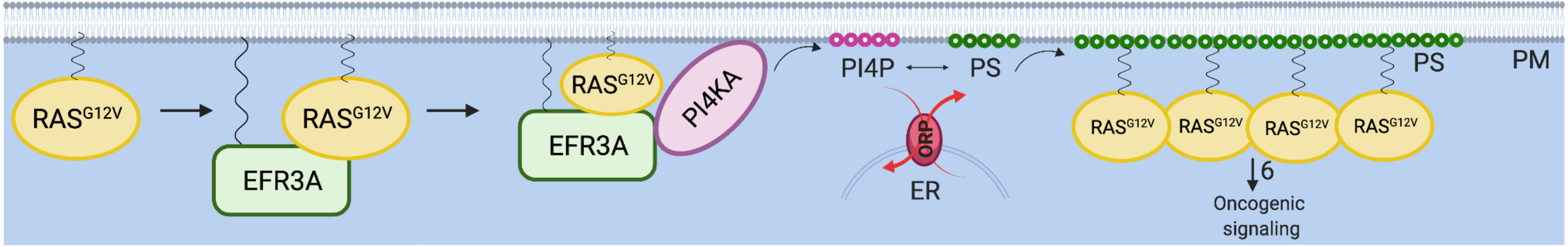

Supplementary Fig. 16. Model of how EFR3A promotes oncogenic RAS signaling. Schematic depicting a model of how EFR3A promotes KRAS-mediated oncogenesis whereby 1) oncogenic KRAS (KRAS^{G12V}) binds EFR3A, 2) EFR3A recruits PI4KA, 3) PI4KA generates PI4P at the plasma membrane (PM). 4) PI4P is exchanged for PS by lipid trans-port proteins, ORP5 and ORP8 at the ER-PM contact sites which, 5) promotes KRAS^{G12V} nanoclustering that then, 6) increases the local concentration of RAS effectors (RAF, PI3K) at the PM to promote oncogenic RAS signaling. Note that PI4P generated by PI4KA is also a substrate for PI3K to generate PI(3,4,5)P3 at the plasma membrane. Created from Biorender.

Supplementary_Fig.17

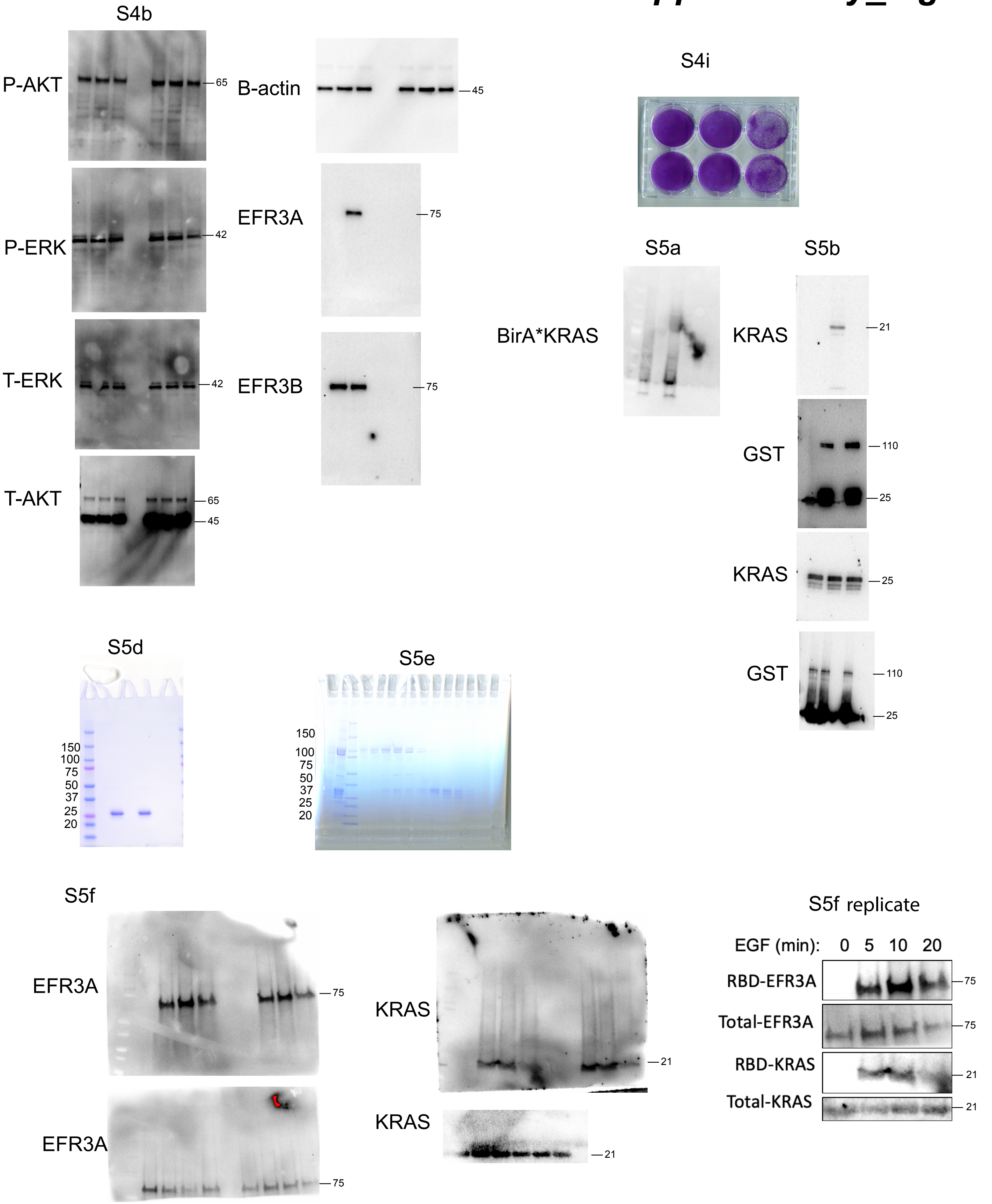

Supplementary Fig. 17. Uncropped images depicted in supplementary figures
