# Supplementary material for: Oncogenic KRAS is dependent upon an EFR3A-PI4KA signaling axis for potent tumorigenic activity
Source: Nat Commun. 2021 Sep 9;12:5248. doi: 10.1038/s41467-021-25523-5 (PMC8429657; doi:10.1038/s41467-021-25523-5)
Supplement: Supplementary file 3 — Reporting Summary [file 41467_2021_25523_MOESM3_ESM.pdf]

nature portfolio

Corresponding author(s): Professor Chris Counter

Last updated by author(s): Jul 28, 2021

Reporting Summary

Nature Portfolio wishes to improve the reproducibility of the work that we publish. This form provides structure for consistency and transparency in reporting. For further information on Nature Portfolio policies, see our Editorial Policies and the Editorial Policy Checklist.

Statistics

For all statistical analyses, confirm that the following items are present in the figure legend, table legend, main text, or Methods section.

n/a	Confirmed	
☐	☒	The exact sample size (*n*) for each experimental group/condition, given as a discrete number and unit of measurement
☐	☒	A statement on whether measurements were taken from distinct samples or whether the same sample was measured repeatedly
☐	☒	The statistical test(s) used AND whether they are one- or two-sided<br>*Only common tests should be described solely by name; describe more complex techniques in the Methods section.*
☐	☒	A description of all covariates tested
☐	☒	A description of any assumptions or corrections, such as tests of normality and adjustment for multiple comparisons
☐	☒	A full description of the statistical parameters including central tendency (e.g. means) or other basic estimates (e.g. regression coefficient) AND variation (e.g. standard deviation) or associated estimates of uncertainty (e.g. confidence intervals)
☐	☒	For null hypothesis testing, the test statistic (e.g. *F*, *t*, *r*) with confidence intervals, effect sizes, degrees of freedom and *P* value noted<br>*Give P values as exact values whenever suitable.*
☒	☐	For Bayesian analysis, information on the choice of priors and Markov chain Monte Carlo settings
☒	☐	For hierarchical and complex designs, identification of the appropriate level for tests and full reporting of outcomes
☒	☐	Estimates of effect sizes (e.g. Cohen's *d*, Pearson's *r*), indicating how they were calculated

Our web collection on statistics for biologists contains articles on many of the points above.

Software and code

Policy information about availability of computer code

Data collection	Commercially available software have been used in the study and described in the methods section of the manuscript. Graphpad prism9.0, ImageJ, 3D Graphing calculator, Synergy finder
Data analysis	Commercially available software have been used in the study and described in the methods section of the manuscript. Graphpad prism9.0, ImageJ, 3D Graphing calculator, Synergy finder

For manuscripts utilizing custom algorithms or software that are central to the research but not yet described in published literature, software must be made available to editors and reviewers. We strongly encourage code deposition in a community repository (e.g. GitHub). See the Nature Portfolio guidelines for submitting code & software for further information.

Data

Policy information about availability of data

All manuscripts must include a data availability statement. This statement should provide the following information, where applicable:

- Accession codes, unique identifiers, or web links for publicly available datasets
- A description of any restrictions on data availability
- For clinical datasets or third party data, please ensure that the statement adheres to our policy

All data have been analyzed using publicly available datasets that can be downloaded at cbioportal database cbioportal.org/datasets as Pancreatic adenocarcinoma [ICGC, Nature 2012; QCMG, Nature 2016; TCGA, Firehose Legacy; TCGA, PanCancer Atlas and for all other cancers, TCGA PanCancer Atlas, datasets for XENA are available at XENA database xenabrowser.net/datapages/ as TCGA Lung Adenocarcinoma, TCGA Bladder Cancer, TCGA Pancreatic Cancer, TCGA Colon Cancer, TCGA Head and Neck Cancer, TCGA Target GTEx) and datasets utilized in GEPIA analysis at gepia.cancer-pku.cn are available as TCGA Pancreatic Cancer dataset at the

XENA database XENAbrowser.net/datapages). Source data are provided with this paper. All the other data are available within the article and its supplementary information.

Field-specific reporting

Please select the one below that is the best fit for your research. If you are not sure, read the appropriate sections before making your selection.

☒ Life sciences ☐ Behavioural & social sciences ☐ Ecological, evolutionary & environmental sciences

For a reference copy of the document with all sections, see nature.com/documents/nr-reporting-summary-flat.pdf

Life sciences study design

All studies must disclose on these points even when the disclosure is negative.

Sample size	Xenograft tumor assays were performed with at least 5 mice per cohort. The in vivo experiments contained 5 mice for each treatment group and the sample size was chosen to reflect a difference in means of 20% with a power of 90%.
Data exclusions	No data has been excluded from the analyses.
Replication	All cell viability assays, soft agar assays have been repeated in triplicate. All immunoblots assay have been repeated in triplicate. All microscopy images have been assessed with at least 30 images. Yes, all replicate experiments were successful.
Randomization	As the experimental groups were equally distributed and hence did not require randomization.
Blinding	Blinding was not performed as every experiment has been validated by multiple approaches to corroborate the research findings. However, the conclusions of hypotheses were tested in multiple experiments by multiple authors. For example, membrane fractionation experiments revealed less KRAS protein levels at the plasma membrane which was again confirmed by electron microscopy. KRAS association with EFR3A have been tested in multiple settings-biochemically,biophysically and by cell biological experiments.

Reporting for specific materials, systems and methods

We require information from authors about some types of materials, experimental systems and methods used in many studies. Here, indicate whether each material, system or method listed is relevant to your study. If you are not sure if a list item applies to your research, read the appropriate section before selecting a response.

Materials & experimental systems

n/a	Involved in the study	
☐	☒	Antibodies
☐	☒	Eukaryotic cell lines
☒	☐	Palaeontology and archaeology
☐	☒	Animals and other organisms
☒	☐	Human research participants
☒	☐	Clinical data
☒	☐	Dual use research of concern

Methods

n/a	Involved in the study	
☒	☐	ChIP-seq
☒	☐	Flow cytometry
☒	☐	MRI-based neuroimaging

Antibodies

Antibodies used	Primary antibodies used detected EFR3A (ThermoFisher Scientific #PA5-54694; diluted 1:100), EFR3B (ThermoFisher Scientific #PA5-107118; diluted 1:250), PI4KA (ThermoFisher Scientific #PA5-28570; diluted 1:50) myc (Cell Signaling #2276; diluted 1:1000), -actin (Cell Signaling #3700; diluted 1:5000), FLAG (Sigma #F3165; diluted 1:1000), KRAS (Sigma #WH0003845M1; diluted 1:1000), P-ERK1/2T202,Y204 (Cell Signaling #9101; diluted 1:1000), P-AKT308 (Cell Signaling #9271; 1:500), AKT (Cell Signaling #9272; diluted 1:1000), ERK (Cell Signaling #9102; diluted 1:1000), BRAF (Cell Signaling #14814; diluted 1:5000), CRAF (Cell signaling #53745; diluted 1:500) and P-CRAF (Cell Signaling #9427; diluted1:500), GST (Santacruz #sc-138; diluted at 1:1000), Membrane fraction WB cocktail (Abcam # : diluted 1:1000) and Strep-HRP (ThermoFisher Scientific # SA10001; diluted 1:20000). Secondary antibodies used were goat anti-rabbit IgG (H+L) HRP (Thermo Fisher Scientific, #65-6120, 1:3000) or goat anti-mouse IgG (H+L) HRP (Life Technologies, #G21040, 1:5000).
Validation	All primary antibodies were validated by using a positive control and negative control sample. For confirmation of knockouts, lentiviruses encoding respective sgRNAs were infected in the cell line of interest, and probed with the respective antibody to validate the absence or presence of bands.

Eukaryotic cell lines

Policy information about cell lines

Cell line source(s)	ATCC or Duke Cell culture Facility. Cell lines for evaluating the loss of EFR3A/B on RAS signaling were created by stably transducing KRASG12V-transformed HEK-HT, HPAF-II, PANC-1, AsPC-1, CFPAC-1, MiaPaCa-2, H358, and H2030 with lentiviruses encoding either vector (Cas9), sgEFR3A-1, sgEFR3A-2, sgEFR3B, or both sgEFR3A-1 and sgEFR3B-1. For co-immunoprecipitation experiments of biotinylated proteins, 293T cells were infected with retroviruses derived from pBabePuro-myc-BirA-KRASG12V, -KRASWT, -or -KRASS17N, after which stable cells were transiently transfected with pcDNA3.1-FLAG-EFR3A plasmid. For visualization of KRAS, HEK-HT cells stably expressing vector (Cas9) or sgEFR3A-1 and sgEFR3B-1 were transduced with lentiviruses encoding GFP-KRASG12V-IRES-mCherry-CAAX. Cells for membrane fractionation studies were generated by stably transducing KRASG12V-transformed HEK-HT, CFPAC-1, and HPAF-II cells with lentiviruses encoding sgEFR3A-1, or both sgEFR3A-1 and sgEFR3B-1. For rescue experiments, KRASG12V-transformed HEK-HTor HPAF-II cells were infected with lentiviruses encoding sgEFR3A-1 and sgEFR3B-1, after which stably-infected cells were infected with retrovirus encoding either pWZL-Neo-myr-myc-KRASG12V-SAAX or pWZL-Neo-myr-FLAG-PI4KA. HEK-HT and MDCK cells were used for electron microscopy
Authentication	All cell lines except MDCK cells were authenticated by STR analysis by Duke Cell culture Facility. MDCK cells have authenticated by STR by the MD Anderson Cancer Center.
Mycoplasma contamination	All cell lines except MDCK cells were tested negative for mycoplasma by Duke Cell culture Facility. MDCK cells were confirmed to be free of mycoplasma contamination at MD Anderson Cancer Center.
Commonly misidentified lines (See ICLAC register)	No misidentified cell lines have been used in the study.

Animals and other organisms

Policy information about studies involving animals; ARRIVE guidelines recommended for reporting animal research

Laboratory animals	Charles River SCID beige mice Strain 250 female mice were used for xenograft tumor assays. The animals were housed in a facility at room temperature with 12 hour dark and light cycle.
Wild animals	No wild animals have been used in the study.
Field-collected samples	No field-collected samples have been used in the study.
Ethics oversight	All animal study experimentally have adhered to the Duke IACUC protocol and policy.

Note that full information on the approval of the study protocol must also be provided in the manuscript.
